# *trans*-Diazido­(1,8-dibenzyl-1,3,6,8,10,13-hexa­azacyclo­tetra­deca­ne)nickel(II)

**DOI:** 10.1107/S1600536808018199

**Published:** 2008-06-19

**Authors:** Jong Won Shin, Kil Sik Min

**Affiliations:** a Department of Chemistry, Kyungpook National University, Daegu 702-701, Republic of Korea; b Department of Chemistry Education, Kyungpook National University, Daegu 702-701, Republic of Korea

## Abstract

In the centrosymmetric title compound, [Ni(N_3_)_2_(C_22_H_34_N_6_)], the Ni^II^ ion is coordinated by the four secondary N atoms of the macrocyclic ligand in a square-planar fashion with two N atoms of the azide ions in axial positions, resulting in a tetra­gonally distorted octa­hedron. An N—H⋯N hydrogen-bonding inter­action between the secondary amine N atom of the macrocycle and an adjacent azide ion gives rise to a chain structure.

## Related literature

For related literature, see: Hancock (1990 ▶); Jacquinot & Hauser (2003 ▶); Jung *et al.* (1989 ▶); Larionova *et al.* (2003 ▶); Min & Suh (2001 ▶); Liu *et al.* (2006 ▶); Tsuge *et al.* (2004 ▶).
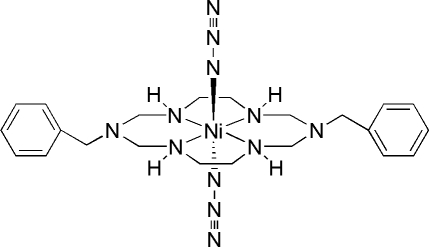


## Experimental

### 

#### Crystal data

[Ni(N_3_)_2_(C_22_H_34_N_6_)]
*M* *_r_* = 525.32
Monoclinic, 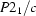

*a* = 10.2150 (5) Å
*b* = 15.8337 (9) Å
*c* = 7.5477 (4) Å
β = 92.817 (1)°
*V* = 1219.30 (11) Å^3^
*Z* = 2
Mo *K*α radiation
μ = 0.83 mm^−1^
*T* = 173 (2) K
0.50 × 0.20 × 0.20 mm

#### Data collection

Siemens SMART CCD diffractometer
Absorption correction: multi-scan (*SADABS*; Sheldrick, 1996 ▶) *T*_min_ = 0.733, *T*_max_ = 0.847
7464 measured reflections
2820 independent reflections
2456 reflections with *I* > 2σ(*I*)
*R*_int_ = 0.020

#### Refinement

*R*[*F* ^2^ > 2σ(*F* ^2^)] = 0.045
*wR*(*F* ^2^) = 0.092
*S* = 1.19
2820 reflections
160 parameters
H-atom parameters constrained
Δρ_max_ = 0.42 e Å^−3^
Δρ_min_ = −0.29 e Å^−3^

### 

Data collection: *SMART* (Siemens, 1996 ▶); cell refinement: *SAINT* (Siemens, 1996 ▶); data reduction: *SAINT* and *SHELXTL* (Sheldrick, 2008 ▶); program(s) used to solve structure: *SHELXS97* (Sheldrick, 2008 ▶); program(s) used to refine structure: *SHELXL97* (Sheldrick, 2008 ▶); molecular graphics: *ORTEP-3* (Farrugia, 1997 ▶); software used to prepare material for publication: *SHELXL97*.

## Supplementary Material

Crystal structure: contains datablocks global, I. DOI: 10.1107/S1600536808018199/er2057sup1.cif

Structure factors: contains datablocks I. DOI: 10.1107/S1600536808018199/er2057Isup2.hkl

Additional supplementary materials:  crystallographic information; 3D view; checkCIF report

## Figures and Tables

**Table 1:** Hydrogen-bond geometry (Å, °)

*D*—H⋯*A*	*D*—H	H⋯*A*	*D*⋯*A*	*D*—H⋯*A*
N3—H3⋯N6^i^	0.93	2.24	3.145 (3)	163

Symmetry code: (i) 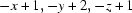.

**Fig. 1.**
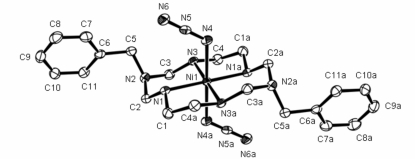
Drawing of the molecular title compound at 50% probability. Atoms labeled with the suffix `a' are at the symmetry position (-x+1, -y+2, -z).

**Fig. 2.**
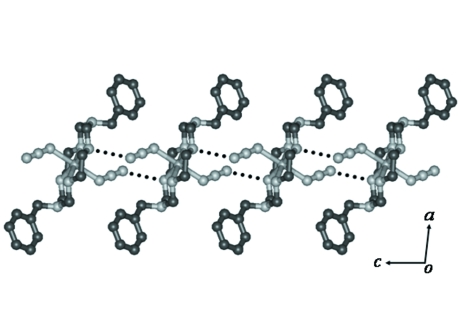
Perspective view of the title compound showing a one-dimensional chain formed by N—H···N hydrogen-bonding interactions.

## supplementary crystallographic information

### Comment

Coordination compounds with tetraaza macrocyclic ligands have been widely studied in the context of metalloenzymes and the construction of extended coordination polymers (Tsuge *et al.*, 2004; Larionova *et al.*, 2003). Especially, Ni^II^ macrocyclic complexes having vacant sites axially are good candidates for assembling novel multi-dimensional networks and catalysts for the reduction of carbon dioxide in which they can have unique properties (Min & Suh, 2001; Jacquinot *et al.*, 2003). Furthermore, the azide ion is a bifunctional ligand which can link to transition metal complexes, thus allowing for the assembly of polymeric compounds (Liu *et al.*, 2006). Therefore, complexes combined with azide ions can also be building blocks for extended network structured materials. Here, we report the synthesis and structure of Ni^II^ macrocyclic complex, *trans*-diazido(1,8-dibenzyl-1,3,6,8,10,13- hexaazacyclotetradecane)nickel(II), with two azide ions axially.

In the title compound, the coordination geometry around Ni^II^ ion is tetragonally elongated octahedron in which Ni^II^ ion is bonded to the four secondary amine N atoms of the macrocyclic ligand in the square-planar fashion and two N atoms from the azide ions at the axial sites as shown in Fig. 1. The average Ni—N_eq_ and Ni—N_ax_ bond distances are 2.072 (1) and 2.159 (1) Å, respectively. The axial Ni—N bond distance is longer than the equatorial Ni—N bond lengths, which can be attributed to the Jahn-Teller distortion of the Ni^II^ ion and/or the ring contraction of the macrocyclic ligand. Two N—N bond distances of the azide ion are not significant different even though one terminal nitrogen atom is coordinated to Ni^II^ ion, indicate that the azide ion is delocalized fully (N4—N5 = 1.189 (3) Å and N5—N6 = 1.171 (3) Å). The complex has an inversion center at the nickel atom and the azamacrocyclic ligand adopts thermodynamically the most stable *R,R,S,S* configuration (Hancock, 1990). All azide ions coordinating Ni^II^ ions axially are involved in hydrogen bonding interactions, which results to a rigid supramolecular one-dimensional chain propagating along the *c* axis (Fig. 2).

### Experimental

The title compound is prepared as follows. To a DMF/H_2_O (*v*/*v*; 1:1, 20 ml) solution of [Ni(C_22_H_34_N_6_)Cl_2_] (0.20 g, 0.40 mmol) (Jung *et al.*, 1989) was added dropwise an aqueous solution (10 ml) containing NaN_3_ (0.052 g, 0.80 mmol) at room temperature. The color of the solution turned from yellow to pale pink. The mixture solution was stirred for 1 h during which time a pink precipitate of formed which was collected by filtration, washed with methanol, and dried in air. Single crystals of the title compound suitable for X-ray crystallography were obtained by layering of the aqueous solution of NaN_3_ on the DMF/H_2_O solution of [Ni(C_22_H_34_N_6_)Cl_2_] for several days.

### Refinement

All H atoms in the title compound were placed in geometrically idealized positions and constrained to ride on their parent atoms, with C—H distances of 0.95 (ring H atoms) or 0.99 (open chain H atoms) Å and N—H distance of 0.93 Å, and with *U*_iso_(H) values of 1.2 times the equivalent anisotropic displacement parameters of the parent C and N atoms.

### Crystal data

**Table:**

[Ni(N_3_)_2_(C_22_H_34_N_6_)]	*F*_000_ = 556
*M**_r_* = 525.32	*D*_x_ = 1.431 Mg m^−^^3^
Monoclinic, *P*2_1_/*c*	Mo *K*α radiation λ = 0.71073 Å
Hall symbol: -P 2ybc	Cell parameters from 2820 reflections
*a* = 10.2150 (5) Å	θ = 2.0–28.3º
*b* = 15.8337 (9) Å	µ = 0.83 mm^−^^1^
*c* = 7.5477 (4) Å	*T* = 173 (2) K
β = 92.817 (1)º	Block, violet
*V* = 1219.30 (11) Å^3^	0.50 × 0.20 × 0.20 mm
*Z* = 2	

### Data collection

**Table:**

Siemens SMART CCD diffractometer	2820 independent reflections
Radiation source: fine-focus sealed tube	2456 reflections with *I* > 2σ(*I*)
Monochromator: graphite	*R*_int_ = 0.020
*T* = 173(2) K	θ_max_ = 28.3º
φ and ω scans	θ_min_ = 2.0º
Absorption correction: multi-scan(SADABS; Sheldrick, 1996)	*h* = −13→11
*T*_min_ = 0.733, *T*_max_ = 0.847	*k* = −17→20
7464 measured reflections	*l* = −9→9

### Refinement

**Table:**

Refinement on *F*^2^	Secondary atom site location: difference Fourier map
Least-squares matrix: full	Hydrogen site location: inferred from neighbouring sites
*R*[*F*^2^ > 2σ(*F*^2^)] = 0.045	H-atom parameters constrained
*wR*(*F*^2^) = 0.092	*w* = 1/[σ^2^(*F*_o_^2^) + (0.0239*P*)^2^ + 1.3575*P*] where *P* = (*F*_o_^2^ + 2*F*_c_^2^)/3
*S* = 1.19	(Δ/σ)_max_ < 0.001
2820 reflections	Δρ_max_ = 0.42 e Å^−^^3^
160 parameters	Δρ_min_ = −0.29 e Å^−^^3^
Primary atom site location: structure-invariant direct methods	Extinction correction: none

### Special details

**Table:**

Geometry. All e.s.d.'s (except the e.s.d. in the dihedral angle between two l.s. planes) are estimated using the full covariance matrix. The cell e.s.d.'s are taken into account individually in the estimation of e.s.d.'s in distances, angles and torsion angles; correlations between e.s.d.'s in cell parameters are only used when they are defined by crystal symmetry. An approximate (isotropic) treatment of cell e.s.d.'s is used for estimating e.s.d.'s involving l.s. planes.
Refinement. Refinement of *F*^2^ against ALL reflections. The weighted *R*-factor *wR* and goodness of fit *S* are based on *F*^2^, conventional *R*-factors *R* are based on *F*, with *F* set to zero for negative *F*^2^. The threshold expression of *F*^2^ > σ(*F*^2^) is used only for calculating *R*-factors(gt) *etc*. and is not relevant to the choice of reflections for refinement. *R*-factors based on *F*^2^ are statistically about twice as large as those based on *F*, and *R*- factors based on ALL data will be even larger.

### Fractional atomic coordinates and isotropic or equivalent isotropic displacement parameters (Å^2^)

**Table:**

	*x*	*y*	*z*	*U*_iso_*/*U*_eq_	
Ni1	0.5000	1.0000	0.0000	0.01702 (11)	
N1	0.37796 (18)	0.89900 (12)	0.0535 (2)	0.0213 (4)	
H1	0.3964	0.8821	0.1701	0.026*	
N2	0.20050 (19)	0.99153 (13)	0.1463 (2)	0.0243 (4)	
N3	0.38842 (18)	1.08789 (12)	0.1271 (2)	0.0204 (4)	
H3	0.4085	1.0836	0.2483	0.024*	
N4	0.6135 (2)	0.97898 (13)	0.2449 (2)	0.0254 (4)	
N5	0.57874 (18)	0.93204 (12)	0.3563 (2)	0.0221 (4)	
N6	0.5460 (2)	0.88500 (14)	0.4654 (3)	0.0315 (5)	
C1	0.4175 (2)	0.83010 (15)	−0.0648 (3)	0.0268 (5)	
H1A	0.3791	0.8395	−0.1862	0.032*	
H1B	0.3852	0.7753	−0.0213	0.032*	
C2	0.2360 (2)	0.92198 (15)	0.0349 (3)	0.0240 (5)	
H2A	0.2139	0.9369	−0.0904	0.029*	
H2B	0.1831	0.8720	0.0644	0.029*	
C3	0.2447 (2)	1.07476 (15)	0.0969 (3)	0.0255 (5)	
H3A	0.1979	1.1175	0.1658	0.031*	
H3B	0.2208	1.0842	−0.0303	0.031*	
C4	0.4333 (2)	1.17143 (15)	0.0675 (3)	0.0258 (5)	
H4A	0.4050	1.2159	0.1495	0.031*	
H4B	0.3948	1.1839	−0.0526	0.031*	
C5	0.2105 (2)	0.97543 (16)	0.3375 (3)	0.0256 (5)	
H5A	0.1964	1.0291	0.4012	0.031*	
H5B	0.3002	0.9555	0.3709	0.031*	
C6	0.1129 (2)	0.91069 (15)	0.3963 (3)	0.0230 (5)	
C7	0.1481 (3)	0.85575 (17)	0.5339 (3)	0.0302 (5)	
H7	0.2352	0.8566	0.5841	0.036*	
C8	0.0580 (3)	0.79974 (17)	0.5991 (3)	0.0359 (6)	
H8	0.0830	0.7633	0.6948	0.043*	
C9	−0.0681 (3)	0.79699 (16)	0.5245 (3)	0.0322 (6)	
H9	−0.1298	0.7582	0.5679	0.039*	
C10	−0.1042 (2)	0.85094 (16)	0.3866 (3)	0.0288 (5)	
H10	−0.1910	0.8491	0.3352	0.035*	
C11	−0.0144 (2)	0.90777 (15)	0.3228 (3)	0.0254 (5)	
H11	−0.0401	0.9448	0.2284	0.031*	

### Atomic displacement parameters (Å^2^)

**Table:**

	*U*^11^	*U*^22^	*U*^33^	*U*^12^	*U*^13^	*U*^23^
Ni1	0.0202 (2)	0.01814 (19)	0.01269 (18)	−0.00082 (17)	0.00037 (13)	0.00017 (16)
N1	0.0243 (10)	0.0257 (10)	0.0140 (8)	−0.0017 (8)	0.0009 (7)	−0.0004 (7)
N2	0.0238 (9)	0.0284 (11)	0.0210 (9)	−0.0012 (8)	0.0040 (7)	−0.0004 (8)
N3	0.0245 (10)	0.0224 (10)	0.0144 (8)	−0.0005 (8)	0.0011 (7)	0.0005 (7)
N4	0.0283 (10)	0.0301 (11)	0.0174 (9)	0.0000 (8)	−0.0017 (8)	0.0042 (8)
N5	0.0216 (10)	0.0257 (10)	0.0187 (9)	0.0035 (8)	−0.0029 (7)	−0.0055 (8)
N6	0.0390 (12)	0.0323 (12)	0.0232 (10)	−0.0042 (10)	0.0017 (9)	0.0044 (9)
C1	0.0291 (13)	0.0229 (12)	0.0287 (12)	−0.0041 (10)	0.0040 (9)	−0.0039 (9)
C2	0.0238 (11)	0.0306 (13)	0.0175 (10)	−0.0050 (10)	0.0010 (8)	−0.0025 (9)
C3	0.0245 (12)	0.0289 (13)	0.0230 (11)	0.0022 (10)	0.0005 (9)	0.0011 (9)
C4	0.0310 (13)	0.0199 (11)	0.0267 (12)	0.0019 (10)	0.0029 (9)	−0.0011 (9)
C5	0.0249 (12)	0.0330 (13)	0.0190 (11)	−0.0031 (10)	0.0019 (9)	−0.0041 (9)
C6	0.0263 (12)	0.0256 (12)	0.0174 (10)	0.0019 (10)	0.0038 (8)	−0.0053 (9)
C7	0.0308 (13)	0.0342 (14)	0.0249 (12)	0.0026 (11)	−0.0037 (10)	0.0015 (10)
C8	0.0493 (16)	0.0319 (14)	0.0263 (13)	0.0015 (12)	0.0006 (11)	0.0077 (10)
C9	0.0386 (15)	0.0278 (13)	0.0312 (13)	−0.0065 (11)	0.0124 (11)	−0.0010 (10)
C10	0.0225 (12)	0.0343 (14)	0.0300 (12)	0.0001 (10)	0.0060 (9)	−0.0056 (10)
C11	0.0238 (12)	0.0283 (12)	0.0243 (11)	0.0053 (10)	0.0033 (9)	0.0008 (9)

### Geometric parameters (Å, °)

**Table:**

Ni1—N3	2.0650 (18)	C2—H2B	0.9900
Ni1—N3^i^	2.0650 (18)	C3—H3A	0.9900
Ni1—N1	2.0795 (19)	C3—H3B	0.9900
Ni1—N1^i^	2.0795 (19)	C4—C1^i^	1.526 (3)
Ni1—N4^i^	2.1592 (19)	C4—H4A	0.9900
Ni1—N4	2.1592 (19)	C4—H4B	0.9900
N1—C1	1.479 (3)	C5—C6	1.512 (3)
N1—C2	1.495 (3)	C5—H5A	0.9900
N1—H1	0.9300	C5—H5B	0.9900
N2—C2	1.443 (3)	C6—C7	1.389 (3)
N2—C3	1.448 (3)	C6—C11	1.389 (3)
N2—C5	1.464 (3)	C7—C8	1.385 (4)
N3—C4	1.477 (3)	C7—H7	0.9500
N3—C3	1.489 (3)	C8—C9	1.381 (4)
N3—H3	0.9300	C8—H8	0.9500
N4—N5	1.189 (3)	C9—C10	1.383 (4)
N5—N6	1.171 (3)	C9—H9	0.9500
C1—C4^i^	1.526 (3)	C10—C11	1.388 (3)
C1—H1A	0.9900	C10—H10	0.9500
C1—H1B	0.9900	C11—H11	0.9500
C2—H2A	0.9900		
			
N3—Ni1—N3^i^	180.0	N1—C2—H2A	108.8
N3—Ni1—N1	94.48 (7)	N2—C2—H2B	108.8
N3^i^—Ni1—N1	85.52 (7)	N1—C2—H2B	108.8
N3—Ni1—N1^i^	85.52 (7)	H2A—C2—H2B	107.7
N3^i^—Ni1—N1^i^	94.48 (7)	N2—C3—N3	113.92 (19)
N1—Ni1—N1^i^	180.0	N2—C3—H3A	108.8
N3—Ni1—N4^i^	90.49 (7)	N3—C3—H3A	108.8
N3^i^—Ni1—N4^i^	89.51 (7)	N2—C3—H3B	108.8
N1—Ni1—N4^i^	89.03 (7)	N3—C3—H3B	108.8
N1^i^—Ni1—N4^i^	90.97 (7)	H3A—C3—H3B	107.7
N3—Ni1—N4	89.51 (7)	N3—C4—C1^i^	108.36 (19)
N3^i^—Ni1—N4	90.49 (7)	N3—C4—H4A	110.0
N1—Ni1—N4	90.97 (7)	C1^i^—C4—H4A	110.0
N1^i^—Ni1—N4	89.03 (7)	N3—C4—H4B	110.0
N4^i^—Ni1—N4	180.00 (10)	C1^i^—C4—H4B	110.0
C1—N1—C2	114.54 (17)	H4A—C4—H4B	108.4
C1—N1—Ni1	105.41 (13)	N2—C5—C6	113.11 (19)
C2—N1—Ni1	112.49 (14)	N2—C5—H5A	109.0
C1—N1—H1	108.1	C6—C5—H5A	109.0
C2—N1—H1	108.1	N2—C5—H5B	109.0
Ni1—N1—H1	108.1	C6—C5—H5B	109.0
C2—N2—C3	117.02 (18)	H5A—C5—H5B	107.8
C2—N2—C5	115.73 (19)	C7—C6—C11	118.8 (2)
C3—N2—C5	113.87 (19)	C7—C6—C5	119.6 (2)
C4—N3—C3	113.36 (18)	C11—C6—C5	121.5 (2)
C4—N3—Ni1	105.99 (13)	C8—C7—C6	121.0 (2)
C3—N3—Ni1	113.40 (14)	C8—C7—H7	119.5
C4—N3—H3	108.0	C6—C7—H7	119.5
C3—N3—H3	108.0	C9—C8—C7	119.9 (2)
Ni1—N3—H3	108.0	C9—C8—H8	120.1
N5—N4—Ni1	122.34 (16)	C7—C8—H8	120.1
N6—N5—N4	179.0 (2)	C8—C9—C10	119.7 (2)
N1—C1—C4^i^	108.84 (19)	C8—C9—H9	120.1
N1—C1—H1A	109.9	C10—C9—H9	120.1
C4^i^—C1—H1A	109.9	C9—C10—C11	120.4 (2)
N1—C1—H1B	109.9	C9—C10—H10	119.8
C4^i^—C1—H1B	109.9	C11—C10—H10	119.8
H1A—C1—H1B	108.3	C10—C11—C6	120.3 (2)
N2—C2—N1	113.67 (18)	C10—C11—H11	119.9
N2—C2—H2A	108.8	C6—C11—H11	119.9
			
N3—Ni1—N1—C1	165.54 (14)	C3—N2—C2—N1	72.2 (3)
N3^i^—Ni1—N1—C1	−14.46 (14)	C5—N2—C2—N1	−66.4 (2)
N4^i^—Ni1—N1—C1	75.12 (14)	C1—N1—C2—N2	−177.66 (18)
N4—Ni1—N1—C1	−104.88 (14)	Ni1—N1—C2—N2	−57.3 (2)
N3—Ni1—N1—C2	40.06 (14)	C2—N2—C3—N3	−71.2 (3)
N3^i^—Ni1—N1—C2	−139.94 (14)	C5—N2—C3—N3	68.1 (2)
N4^i^—Ni1—N1—C2	−50.35 (14)	C4—N3—C3—N2	176.97 (18)
N4—Ni1—N1—C2	129.65 (14)	Ni1—N3—C3—N2	56.1 (2)
N1—Ni1—N3—C4	−164.63 (14)	C3—N3—C4—C1^i^	−166.97 (18)
N1^i^—Ni1—N3—C4	15.37 (14)	Ni1—N3—C4—C1^i^	−41.96 (19)
N4^i^—Ni1—N3—C4	−75.56 (14)	C2—N2—C5—C6	−66.6 (3)
N4—Ni1—N3—C4	104.44 (14)	C3—N2—C5—C6	153.5 (2)
N1—Ni1—N3—C3	−39.65 (15)	N2—C5—C6—C7	145.0 (2)
N1^i^—Ni1—N3—C3	140.35 (15)	N2—C5—C6—C11	−38.7 (3)
N4^i^—Ni1—N3—C3	49.42 (15)	C11—C6—C7—C8	−0.9 (4)
N4—Ni1—N3—C3	−130.58 (15)	C5—C6—C7—C8	175.5 (2)
N3—Ni1—N4—N5	83.63 (19)	C6—C7—C8—C9	1.2 (4)
N3^i^—Ni1—N4—N5	−96.37 (19)	C7—C8—C9—C10	−0.8 (4)
N1—Ni1—N4—N5	−10.84 (19)	C8—C9—C10—C11	0.0 (4)
N1^i^—Ni1—N4—N5	169.16 (19)	C9—C10—C11—C6	0.3 (4)
C2—N1—C1—C4^i^	165.51 (18)	C7—C6—C11—C10	0.1 (3)
Ni1—N1—C1—C4^i^	41.3 (2)	C5—C6—C11—C10	−176.2 (2)

Symmetry codes: (i) −*x*+1, −*y*+2, −*z*.

### Hydrogen-bond geometry (Å, °)

**Table:**

*D*—H···*A*	*D*—H	H···*A*	*D*···*A*	*D*—H···*A*
N3—H3···N6^ii^	0.93	2.24	3.145 (3)	163

Symmetry codes: (ii) −*x*+1, −*y*+2, −*z*+1.
